# Heterologous Ferredoxin Reductase and Flavodoxin Protect Cos-7 Cells from Oxidative Stress

**DOI:** 10.1371/journal.pone.0013501

**Published:** 2010-10-19

**Authors:** María G. Mediavilla, Gisela A. Di Venanzio, Edgardo E. Guibert, Claudio Tiribelli

**Affiliations:** 1 Centro Binacional (Argentina-Italia) de Investigaciones en Criobiología Clínica y Aplicada (CAIC) and Consejo Nacional de Investigaciones Científicas y Técnicas (CONICET), Rosario, Argentina; 2 Área Biología Molecular, Facultad de Ciencias Bioquímicas y Farmacéuticas, Universidad Nacional de Rosario, Rosario, Argentina; 3 Centro Studi Fegato, AREA Science Park, Basovizza, and Department ACADEM, University of Trieste, Trieste, Italy; Universidade de Brasília, Brazil

## Abstract

**Background:**

Ferredoxin-NADP(H) reductase (FNR) from *Pisum sativum* and Flavodoxin (Fld) from *Anabaena* PCC 7119 have been reported to protect a variety of cells and organisms from oxidative insults. In this work, these two proteins were expressed in mitochondria of Cos-7 cells and tested for their efficacy to protect these cells from oxidative stress *in vitro*.

**Principal Findings:**

Cos-7/pFNR and Cos-7/pFld cell lines expressing FNR and Fld, respectively, showed a significantly higher resistance to 24 h exposure to 300–600 µM hydrogen peroxide measured by LDH retention, MTT reduction, malondialdehyde (MDA) levels and lipid peroxide (LPO; FOX assay) levels. However, FNR and Fld did not exhibit any protection at shorter incubation times (2 h and 4 h) to 4 mM hydrogen peroxide or to a 48 h exposure to 300 µM methyl viologen. We found enhanced methyl viologen damage exerted by FNR that may be due to depletion of NADPH pools through NADPH-MV diaphorase activity as previously observed for other overexpressed enzymes.

**Significance:**

The results presented are a first report of antioxidant function of these heterologous enzymes of vegetal and cyanobacterial origin in mammalian cells.

## Introduction

Ferredoxin-NADP(H) reductases (FNRs; EC 1.18.1.2) constitute a family of hydrophilic, monomeric enzymes that contain non-covalently bound flavin adenine dinucleotide (FAD) as a prosthetic group. These ubiquitous flavoenzymes are present in animals, plants, parasites and prokaryotes, where they catalyze the reversible exchange of electrons between two molecules of a variety of obligatory one-electron carriers, such as ferredoxins (Fds) and flavodoxins (Flds), and a single molecule of NADP(H) [1]. Although FNRs are present in all kingdoms, plant isoforms are 200–500 times more active than their animal or prokaryote counterparts [1], property that could explain their notable competency as antioxidants in bacterial models [2], [3]. FNRs have demonstrated to protect proteobacteria and cyanobacteria from oxidative stress [2]–[5]. *Pisum sativum* (pea) FNR accomplishes functional complementation of mutant *Escherichia coli* defective for *mrvA* gene that are unable to grow aerobically in the presence of the radical propagator methyl viologen (MV: 1,1′-dimethyl-4,4′-bipyridinium dichloride) [4]. This complementation is strictly dependent on accumulation of functional transgenic FNR [4]. The NADP(H) dependent activities of the reductase were necessary and sufficient for detoxification, without participation of either Fd or Fld in the process [2]. Transgenic tobacco plants expressing knocked down levels of FNR are abnormally prone to photo-oxidative injury [6]. When grown under autotrophic conditions for 3 weeks, specimens with 20–40% extant reductase underwent leaf bleaching, elevated malondialdehyde (MDA) levels and membrane damage [6].

Given these antecedents, we hypothesized that pea FNR could be beneficial in the protection of mammalian cells from the oxidative injury produced by cold ischemia/reperfusion. In a previous work [7], we applied FNR gene transfection to isolated hepatocytes preserved in University of Wisconsin solution and then transplanted into recipient animals. We observed that, after cold preservation, transplanted hepatocytes expressing pea FNR were found in the parenchyma of recipient rat livers in quantities 20–50 times greater than controls and were devoid of the surrounding inflammatory infiltrates normally present when hepatocyte engraftment occurs. These results were indicative of an advantage of FNR-expressing cells during implantation. Two facts were already known: 1- donor hepatocytes show a burst of reactive oxygen species (ROS) soon after transplantation (cold ischemia-reperfusion injury) [8]–[12]; and 2- the obstruction of sinusoids produced by donor hepatocyte arrival through portal circulation recruits Kuppfer cells that exert their local action by releasing of ROS [13], [14]. Consequently, it seemed that FNR transfected hepatocytes could overcome these challenges better and engraft more effectively in the receptor liver than control cells [7]. Since our original hypothesis was that an advantage of FNR-expressing hepatocytes should be due to an antioxidant effect of this transgene, we became interested to assess whether pea FNR protects mammalian cells from oxidative insults in a less complex *in vitro* model that allowed a direct challenge to oxidants and non-ambiguous interpretation of the results.

Flavodoxins, on the other hand, are small acidic proteins that transfer electrons at low potentials and contain a non-covalently bound flavin mononucleotide (FMN) as their sole redox center. They act in various electron transport systems as functional analogs of Fds. Despite the fact that they have been found only in bacteria and algae, they share similarity with a number of protein domains of both prokaryote and eukaryote origin [15]. In cyanobacteria and enterobacteria, Fld levels increase several fold on exposure to MV and other superoxide (O_2_^•-^)-propagating compounds [5], [16], [17], and overexpression of the flavoprotein in *E. coli* leads to augmented tolerance toward various sources of oxidative stress [16]. In transgenic tobacco plants, expressing *Anabaena* Fld in a constitutive fashion, this protein has exerted a notable protection against photo-oxidative and hydric stresses [18]. As in the case of FNR, Fld seems a good candidate for exploration of its protective antioxidative properties in new systems as mammalian cells are.

In this work we attempted the evaluation of the capacity of *P. sativum* FNR and *Anabaena* PCC 7119 Fld to protect Cos-7 cells from oxidative stress challenges *in vitro*. We found that both FNR and Fld protected against hydrogen peroxide (H_2_O_2_) after 24 hours of exposure. Surprisingly, we did not observe protection towards MV by neither of these transgenic proteins.

These results reveal antioxidant functions of plant FNR and cyanobacterial Fld in a mammalian cell line opening the opportunity to examine a myriad of applications in diseases where reactive oxygen species (ROS) are know to play a role in etiology (cancer, Alzheimer) and other clinical situations such as transplantation where the involvement of ROS in primary organ failure has been recognized.

## Materials and Methods

### Genes and plasmids

A full length cDNA (1385 nt; GenBank Accession Number X12446) of *Pisum sativum* FNR gene was cloned from a pea library [19]. *Anabaena* Fld gene sequence (540 nt) [20] was obtained by PCR cloning from genomic DNA (GenBank Accession Number S68006). A full length cDNA (1898 nt; GenBank Accession Number D49920) coding for mouse ferredoxin reductase (Fdxr) gene was cloned from a mouse kidney library [21].

To construct pFNR (Figure S1) plasmids p-FR7-1 [21] and pCV105 [22] were used to obtain the sequences of mitochondrial localization signal (MLS) of mouse Fdxr and mature portion of pea FNR genes, respectively. These sequences were fused in frame and inserted into commercial plasmid pcDNA3 (Invitrogen V79020) between the *Hind* III and *Eco* RI sites of its multiple cloning site.

To generate plasmid pFld (Figure S2) the sequence of Fld gene from *Anabaena* PCC 7119 [20], in plasmid pFlavo, was amplified by PCR introducing *Sac* I and *Eco* RI restriction sites flanking the open reading frame, fused in frame with MLS of mouse Fdxr gene [21], and inserted into pcDNA3.

pFNR and pFld sequences were checked in a Hitachi 3100 Avant Genetic Analyzer (Applied Biosystems) using the Big Dye kit (Applied Biosystems 4336697 and 4336768) and Avant Data Collection Software (Applied Biosystems) (Figure S3).

### Cell culture and transfections

Cos-7 cells (ECACC 87021302) were cultured in DMEM/High glucose (Sigma D6546) supplemented with 2 mM L-glutamine (EuroClone EC B3000D) and 10% fetal bovine serum (FBS, Sigma F7524) in a CO_2_ incubator at 37°C. Transfections were done using Lipofectamine 2000 (Invitrogen 11668-027) following manufacturer's instructions. Transfectant cells were selected with 0.50 mg/ml G418 (Sigma A1720) for 15 days until complete detachment of mock-transfected cells. The G418 concentration for selection was established by performing a killing curve on Cos-7 cells for 2 weeks. To conduct the experiments, all lines were used within 20 passage numbers to reduce variations due to cell line characteristic alterations.

### Evaluation of transgene expression and cellular localization

For RT-Real Time PCR total cellular RNA was isolated using TRI-REAGENT (Sigma T9424) and then treated with DNAse. Quantification and quality evaluation were performed spectrophotometrically and integrity was assessed in agarose-formaldehyde gels. RNA was retrotranscribed into cDNA with the commercial kit iScript (Bio-Rad 170-8890). Primers (FNR FP: 5′TGGTTTGGCATGGCTCTTCC3′; FNR RP: 5′ATCGTTTACTTGCTCTCTGCTTAC3′; Fld FP: 5′TTGATTATTGGCTGTCCTACTTGG3′; Fld RP: 5′CTGCGTAACCTATTTGGTCACC3′) were designed with the aid of Beacon Designer 2.0 (PREMIER Biosoft International). Real Time PCR was performed using iQ SYBR Green Supermix (Bio-Rad 170-8882) in Gene Amp PCR System 2400 thermocycler (Perkin-Elmer) controlled by i-Cycler IQ program (Bio-Rad). Negative controls using non-retrotranscribed RNA were included and the amplification products were checked in agarose gels after Real Time-PCR completion.

The sub-cellular localization of the transgenic proteins was verified. Total proteins were extracted with 20 mM Tris/HCl pH 7.50, 150 mM NaCl, 1 mM EDTA, 1 mM EGTA, 1% Triton X-100, 1 mM Na_3_VO_4_, 1 mM PMSF and 10 µl/ml protease inhibitor cocktail (Calbiochem 539137), directly from the culture plate. Cytosolic, mitochondrial and nuclear protein fractions were obtained by a differential centrifugation protocol. Briefly, cells (approximately 30×10^6^ cells) were detached from culture surface, mechanically disrupted by several passages trough a 25G syringe avoiding foam formation and debris were eliminated by low speed centrifugation (500 *g*). Nuclear and mitochondrial enriched fractions were obtained centrifuging at 2,500 *g* and 10,000 *g*, respectively, and were further washed and purified by more stringent centrifugation speeds. Cytosolic fraction was cleared by high speed centrifugation. Enrichment and cross-contamination of fractions were determined measuring the activities of the classical organelle marker enzymes: citrate synthase, NADH:ubiquinone oxidoreductase, lactate deshydrogenase, glucose-6-phosphatase, catalase and acid phosphatase as previously reported [23]–[25]. Fractions were probed for transgenic protein presence by standard western blotting (WB) using rabbit anti-FNR and rabbit anti-Fld antibodies and polyclonal swine anti-rabbit IgG/HRP (DakoCytomation P0217). Immunofluorescence (IF) co-localization studies were performed by labeling with the same primary antibodies and fluorescein anti-rabbit IgG (Vector FI-1000); and Mitotracker Red 580 (Molecular Probes M22425) for mitochondrial staining. Images were taken with an inverted confocal microscope (Nikon C1/Eclipse TE-2000-E2). Pearson's Correlation (Rr) and Overlap (R) Coefficients were also calculated (EZ-C1 3.70 software, Nikon).

Diaphorase activity for FNR was measured as the reduction of ferricyanide in a reaction mixture (1 ml) containing 50 mM Tris-HCl pH 8.50, 5 mM MgCl_2_, 3 mM glucose-6-phosphate, 300 µM NADP^+^, 1 U glucose-6-phosphate dehydrogenase and 1 mM potassium ferricyanide. The decrease in absorption at λ = 420 nm was monitored at 30°C using an ε_420_ = 1 mM^−1^ cm^−1^ [26].

### Oxidative stress induction

Cells were plated 24 h before treatment at a density of 25,000 cells/cm^2^ and then exposed to 0–4 mM H_2_O_2_ (Sigma H1009) for 2 and 4 h, and to 0–600 µM H_2_O_2_ for 24 h in complete medium. For MV (Aldrich 85617-7) incubation cells were plated 24 h before treatment at a density of 12,500 cells/cm^2^. Cells were then exposed to 0–300 µM MV in complete medium for 48 h.

### Cell viability and metabolic function

LDH activity was determined by measuring NADH oxidation at λ = 340 nm in a reaction mixture containing 0.6 mM pyruvate and 0.2 mM NADH in 50 mM KPO_4_ pH 7.50. Δ*A*/min was monitored for 3 min at 37°C.

For MTT (3-[4,5-dimethylthiazol-2-yl]-2,5-diphenyl-tetrazolium bromide) reduction assay cell monolayers were washed with PBS and 0.50 mg/ml MTT (Sigma M2128) in culture medium was added and incubated for 2 h in a CO_2_ incubator. After incubation, insoluble violet formazan crystals were dissolved with 3% SDS and 0.03 N HCl in 70% isopropanol and absorbance at λ = 570 nm was read.

### Oxidative damage to lipids

Fe^3+^/xylenol orange (XO: 1-methyl-1-phenylethylhydroperoxide sodium salt) complex formation due to lipid peroxides present in the sample (FOX assay) was determined [27], [28]. Monolayers were washed and incubated with 100 µM XO (Aldrich 513296), 250 µM Fe (NH4)_2_ (SO4)_2_ and 110 mM HClO_4_ for 30 min at room temperature. Absorbance was then read at λ = 560 nm.

Malondialdehyde (MDA) quantification was performed in an HPLC system using a C18 column. Mobil phase consisted in 30 mM KH_2_PO_4_ pH 4.00/methanol (65/35% v/v) at a flow rate of 1.5 ml/min [29]. UV detection was set at λ = 254 nm. As standard, TEP (1,1,3,3-tetraethoxypropane, Sigma T9889) was diluted in 0.1 M HCl, boiled for 5 min and diluted to 50 µM MDA (ε = 13700 M^−1^ cm^−1^ at λ = 254 nm). Samples and standard were diluted (1/20) in cold HClO_4_ (final concentration 25 mM), filtered and injected immediately into the column (20 µl). Culture supernatants were used as it was shown that MDA is almost completely released to culture medium within 1 h after production [30].

### Data analysis

All the data were expressed as percentage of change with respect each cell line not exposed to H_2_O_2_ or MV (control cultures, 100%). Results are expressed as mean ± standard deviations (SD). Experiments were performed in triplicate and its number (n) is reported in the figures. Groups of data were compared by analysis of variance (ANOVA) followed by Tukey post test. Statistical analyses were performed by InStat 3.05 (GraphPad Software Inc.) free software.

## Results

### Cell lines

Three new cell lines were generated by transfection with pcDNA3, pFNR and pFld plasmids and named Cos-7/pcDNA3, Cos-7/pFNR and Cos-7/pFld, respectively. They showed conserved epithelial cell morphology and no evident changes in shape or size (cellular or nuclear) with respect to original Cos-7 cells. Rate of growth was also similar in the absence of G418 antibiotic but to some extent slower in its presence (Figure S4). Based on this evidence, the results obtained have been normalized to controls performed with the same cell line.

### Evaluation of transgene expression and cellular localization

We confirmed by RT-PCR that Cos-7 and Cos-7/pcDNA3 do not express the analyzed messengers, Cos-7/pFNR express only pea FNR and Cos-7/pFld express only *Anabaena* Fld. These results were confirmed and expanded by WB and IF were the cellular localization of FNR in Cos-7/pFNR and Fld in Cos-7/pFld was assessed (Figure S5). For the FNR detection in WB two positive bands were seen in Cos-7/pFNR total extract and mitochondrial fraction lanes; no bands were revealed in the lanes corresponding to total extracts of Cos-7, Cos-7/pcDNA3 and Cos-7/pFld or cytoplasmic and nuclear fractions of Cos-7/pFNR cells (Figure S5 A). The major band showed a *Mr* of 35.76 kDa identical to the expected one (35.34 kDa) for the processed form of the enzyme after cleavage of the MLS at the predicted site. The minor band presented a *Mr* of 43.23 kDa somewhat higher than calculated for the entire protein (39.72 kDa). Co-localization estimations gave Rr = [0.571-0.846] and R = [0.977-0.985] (n = 10 cells for each of 2 independent staining analyses) pointing to a high degree of overlap between mitochondria and FNR signals (Figure S5 B). Similar studies were performed for Fld. Again, positive bands in WB were only observed for total extract and mitochondrial fraction of Cos-7/pFld while all other lines and fractions resulted negative (Figure S5 A). Also in this case two bands were found: a major band of 33.24 kDa (higher than 24.84 kDa expected for the whole protein) and a minor one of 19.87 kDa very similar in *Mr* to the predicted one (20.46 kDa) for the processed protein lacking the MLS. By IF (Figure S5 B) overlap coefficients Rr = [0.602-0.741] and R = [0.973-0.982] were found (n = 10 cells for each of 2 independent staining analyses) indicating elevated co-localization between Fld and mitochondria.

Cos-7/pFNR total diaphorase activity was almost twice of that of Cos-7/pcDNA3 (49.6±1.6 mU/mg prot *vs.* 27.1±4.3 mUI/mg prot, p<0.0001).

### Hydrogen peroxide cytotoxicity

**Figure 1:**
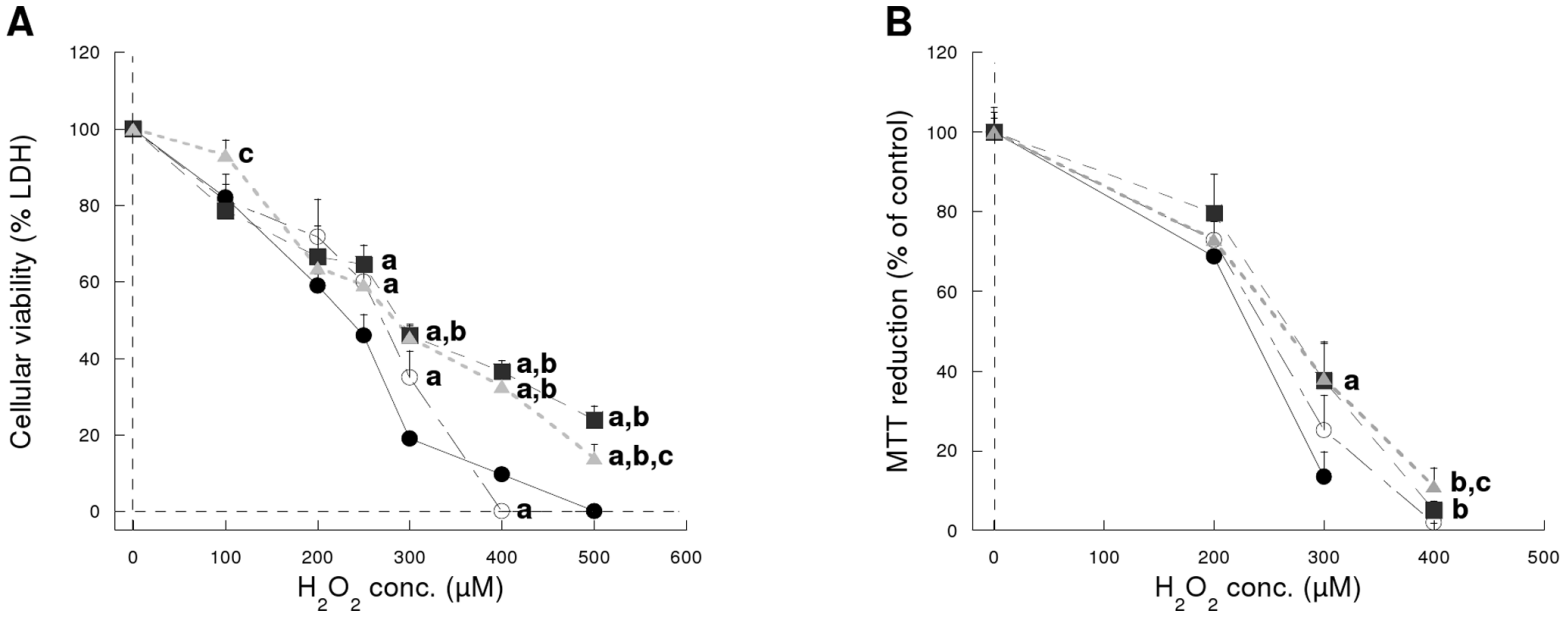
Cytotoxicity after 24 h exposure to H_2_O_2_. (**A**) Cellular viability measure as LDH retention (n = 4 experiments, in triplicate). (**B**) Cellular metabolism measured as the reduction of MTT (n = 12 experiments, in triplicate). (•) Cos-7 cells, (○) Cos-7/pcDNA3 cells (▪), Cos-7/pFNR cells and (▴) Cos-7/pFld cells. **^a^** different from Cos-7 (p<0.05), **^b^** different from Cos-7/pcDNA3 (p<0.05), **^c^** different from Cos-7/pFNR (p<0.05).

Figure 1 shows the effects of 24 h incubation with H_2_O_2_ on the cell viability and MTT test. Both FNR and Fld showed a clear protective effect (p<0.0001 by ANOVA) at H_2_O_2_ concentrations higher than 300 µM as evidenced by the difference with Cos-7 and Cos-7/pcDNA3 control cells. No protection was observed at 2 and 4 h incubation time even thought at the highest H_2_O_2_ concentration tested (4 mM), cell viability was reduced to 40% and 10%, respectively (Figure S6).

**Figure 2:**
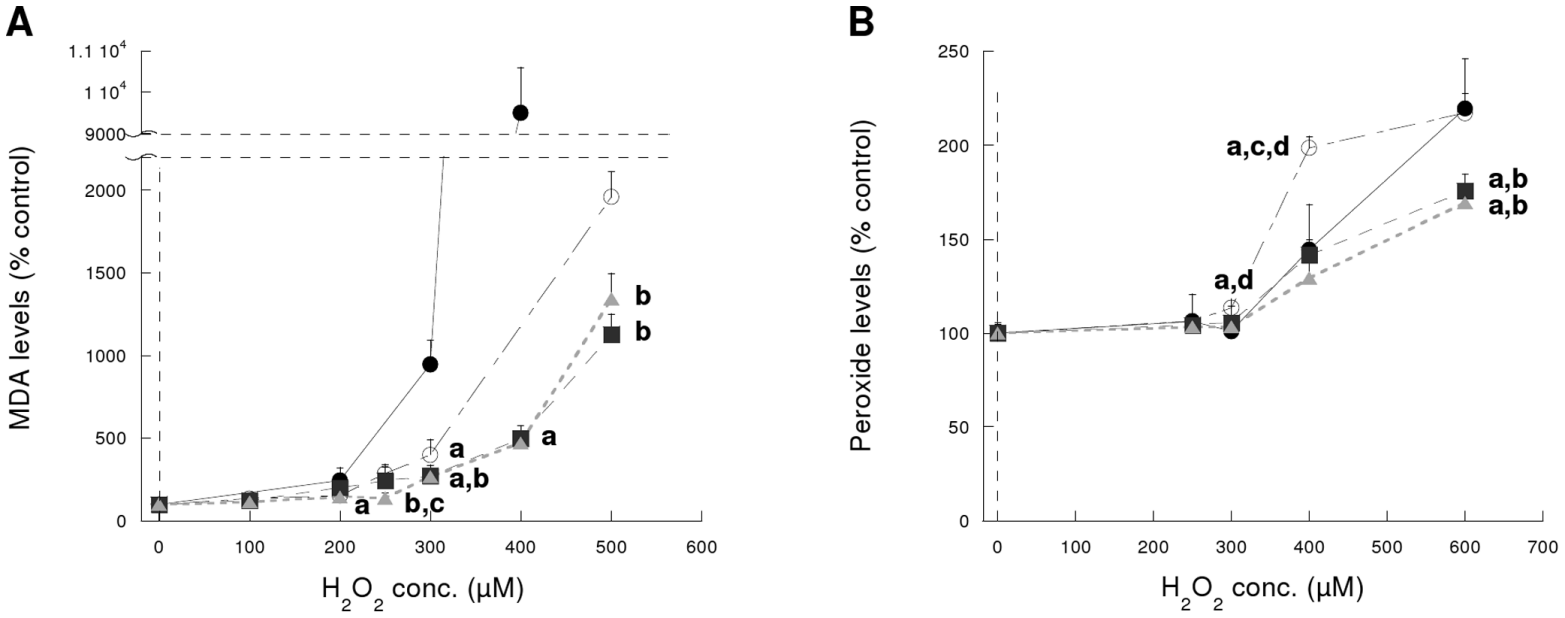
Oxidative damage to lipids after 24 h exposure to H_2_O_2_. (**A**) MDA levels. (**B**) Peroxide levels (FOX assay). (•) Cos-7 cells, (○) Cos-7/pcDNA3 cells, (▪) Cos-7/pFNR cells and (▴) Cos-7/pFld cells. **^a^** different from Cos-7 (p<0.05), **^b^** different from Cos-7/pcDNA3 (p<0.05), **^c^** different from Cos-7/pFNR (p<0.05), **^d^** different from Cos-7/pFld (p<0.05). n = 4 experiments, in triplicate.

With regard to the measurement of the oxidative damage to lipids, a similar protection was observed after an exposure of 24 h (Figure 2) since both MDA and lipoperoxides levels were significantly lower (p<0.001 by ANOVA) than in control lines. After 4 h incubation lipid peroxide levels (FOX assay) did not differ from untreated controls or between cell lines (not shown).

### Methyl viologen cytotoxicity

**Figure 3:**
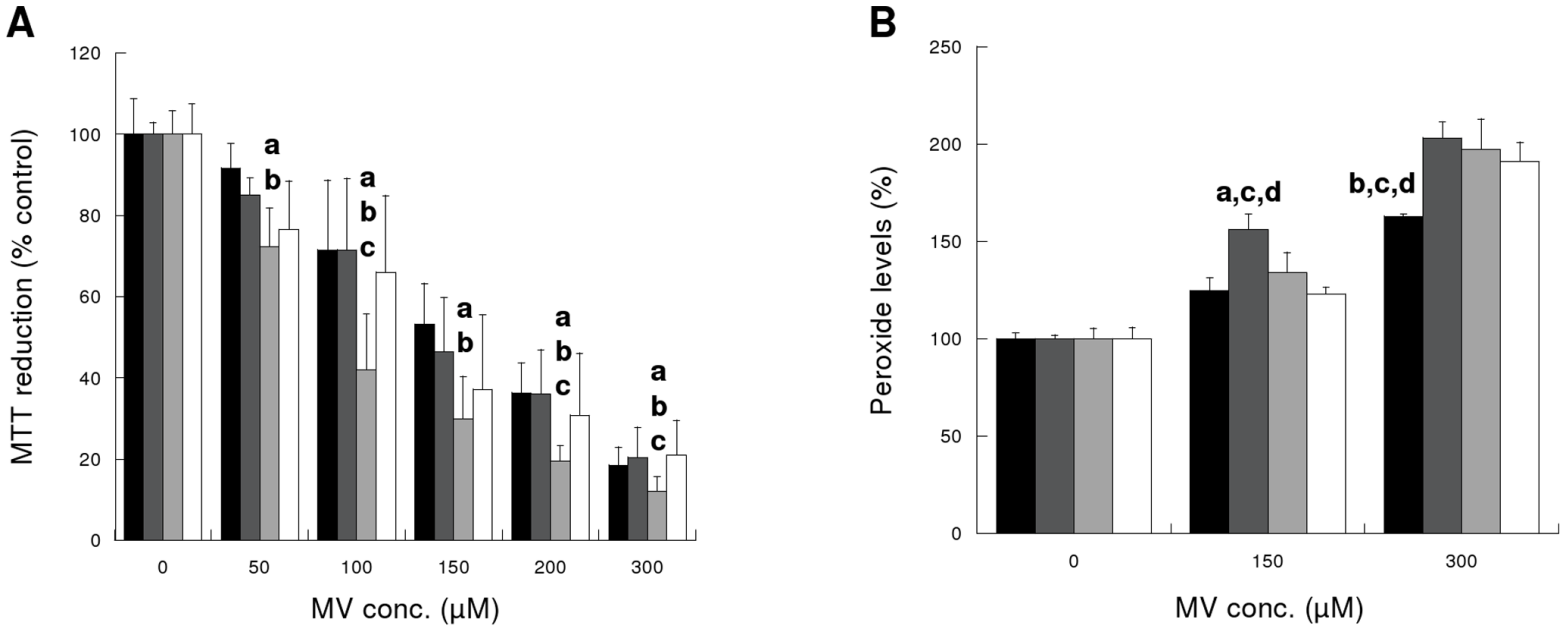
Cytotoxicity after 48 h exposure to MV. (**A**) Cellular metabolism (MTT reduction). (**B**) Lipid peroxidation levels (FOX assay). Black bars: Cos-7 cells, dark grey bars: Cos-7/pcDNA3 cells, light grey bars: Cos-7/pFNR cells and white bars: Cos-7/pFld cells. **^a^** different from Cos-7 (p<0.05), **^b^** different from Cos-7/pcDNA3 (p<0.05), **^c^** different from Cos-7/pFNR (p<0.05), **^d^** different from Cos-7/pFld (p<0.05). n = 4 experiments, in triplicate.

We could not find any protection exerted by FNR or Fld against MV injury as measured by MTT reduction and FOX assay (Figures 3A and B, respectively). Moreover, Cos-7/pFNR resulted to be more susceptible to injury than the other cell lines (Figure 3A).

## Discussion

In this work we investigated the capacity of *P. sativum* FNR and *Anabaena* Fld to protect Cos-7 cells from oxidative stress induced by hydrogen peroxide and methyl viologen.

Previous work performed by various groups demonstrated the successful interactions and electron transfer of the components of hybrid systems as *Anabaena* FNR and bovine adrenodoxin [31] and bovine adrenodoxin reductase and Anabaena PCC 7119 Fld [32]. It was also shown that NADPH-Fld reductase/Fld from *E. coli* can function as electron donors to bovine P450c17 to the same proportional extent (hydrolase to lyase ratio) as does P450 reductase [33]. NADPH-cytochrome P-450 oxidoreductase gene has most probably arisen through the fusion of the ancestral genes of Fld and FNR [15]. All these evidences prompted us to undertake the present work to test the hypothesis that heterologous proteins originating from plant and cyanobacteria would still be functional in Cos-7 cells.

Both proteins were directed to mitochondria via the fusion to their sequence of a MLS of mouse ferredoxin reductase. A number of works reported that proteins can be successfully directed to mitochondria by just fusing their N-terminal ends to a MLS [34]–[37]. Mouse Fdxr MLS was recognized by Cos-7 cells where it effectively directed both FNR and Fld to mitochondria. For FNR the MLS seems to be processed correctly as shown by WB analysis were we found a major band corresponding to the size of the mature protein and with only a small proportion remaining as the full-length, non-processed form. This is different for Cos-7/pFld cells where the proportion of the processed form was smaller indicating that the MLS have been cleaved less effectively. This can be explained taking into account that Fld does not posses a localization signal in its native form while pea FNR complete gene bears a chloroplast localization signal making its mature portion sequence appropriate for enzymatic digestion after transport. The exact position at which the cleavages of FNR and Fld are accomplished by Cos-7 cells is still to be determined.

Mitochondria have clearly been recognized as the most important physiological source of ROS being, at the same time, a cellular target for free radical-mediated damage as described for a series of diseases and during aging. Mitochondria are also the origin place of signaling molecules that command cell cycle, proliferation and apoptosis. Conservation of the mitochondrial DNA and respiratory chain components integrity is of most importance as they are the main source of cell energy production through oxidative phosphorylation. If antioxidant defense mechanisms are compromised or overwhelmed for any reason, a disease can arise as for example neurodegenerative disorders, cancer or aging [38]. In mammalian cell mitochondria, H_2_O_2_ provokes the disruption of the permeability transition with the consequent induction of apoptosis [39]. The main defense system against H_2_O_2_ is constituted by the glutathione redox cycle and Glutathione Peroxidase activities [39] together with Mn-SOD and the reductants NADH_2_ and UQH_2_. Recently, it has been demonstrated that MV accumulates in mitochondria through a carrier-mediated transport which requires intact membrane potential and, once inside, increases intramitochondrial O_2_^•-^ production [40]. Reduction and autooxidation of MV outside the mitochondria seemed to be negligible [40]. In mammalian mitochondria, different from other organisms, MV is mainly reduced by Complex I by reverse electron transport [40]. For these reasons, we considered mitochondria an appropriate cellular site to express FNR and Fld as a first approximation to examine their actions.

After 2 and 4 h exposure to H_2_O_2_ at concentrations above 1.5 mM, we observed a marked lost of metabolic function as measured by MTT reduction (Figure S6 A) for all cell lines with no protection from FNR and Fld. The lack of protection is probably due to the high concentration of H_2_O_2_ used which has been reported to induce a rapid death by necrosis [41]. These H_2_O_2_ concentrations were chosen because they allowed to measure a reduction of MTT detoxification at the short-time exposures studied (2 and 4 h).

Cellular viability (LDH release) and metabolism (MTT test) were protected by both FNR and Fld towards exposure to hydrogen peroxide for 24 h at concentrations of 300 µM and above (Figure 1). It is known that cells can display different responses to cytotoxicity injuries going from quiescence to apoptosis and necrosis, with different degrees in between. It has been reported that at H_2_O_2_ concentrations of 250–400 µM cells enter a “permanently growth-arrested state”, sometimes confused with cell death in toxicity studies, while at higher concentrations (0.5–1.0 mM) they will enter the apoptotic pathway [41]. The range of concentrations at which FNR and Fld display protection in this work overlap both situations described and thereby it is possible that the two phenomena are occurring with cell death gaining importance at the higher concentrations tested.

Hydrogen peroxide preferentially partitions into lipid and hydrophobic cores of proteins. The oxidative biomarker hypothesis proposes the theory that measurement of levels of oxidized biomolecules can provide an index of the levels of oxidant to which a biological system has been exposed [42]. Cells expressing FNR and Fld showed reduced oxidative damage of lipids (Figure 2) indicating that the protection is probably exerted through an antioxidative action towards H_2_O_2_. Even though a mechanism of protection through repair can not be discarded and it has been observed for iron-sulfur centers of hydro-lyases in FNR-overexpressing bacterial cells after oxidative stress induction [43], FNR and Fld are not repairing enzymes *per se*. However, it is possible that some repairing pathways are working more efficiently in cells expressing FNR and Fld because they are more protected by the actions of these two proteins. Moreover, even though low H_2_O_2_ concentrations (120–150 µM) are known to be rapidly metabolized by cells and provoke a reversible cellular arrest that last only 2 to 4 h [41], Kang et al. [44] have found several fold increased ROS levels in their Cos-7-pcDNA3.0 cells (equivalent to Cos-7/pcDNA3 in this work) after 24 h incubation with 250 and 500 µM H_2_O_2_. This observation reinforces the hypothesis that oxidative stress persists 24 h after induction by hydrogen peroxide.

MDA levels were much higher for Cos-7 than Cos-7/pcDNA3 cells. This difference is not really surprising as Cos-7 cell line has a faster rate of growth than all the transfected lines when grown in the presence of G418 (Figure S4). Faster growing cells are metabolically more active and, as a consequence, more susceptible to damages by exposure to cytotoxic compounds. Given this observation, we considered important to include both lines (Cos-7 and Cos-7/pcDNA3) as controls in this study to be able to discriminate between the effects of the expression of FNR and Fld *per se* (when compared with Cos-7/pcDNA3) and including other factors of the process of transfection and selection (when compared with Cos-7). Both controls are necessary to allow comparison in the case transfection or transduction systems different from the one used here were employed.

The protection from hydrogen peroxide-induced damage resulted of similar magnitude for FNR and Fld. This finding was not expected *a priori* because FNR and Fld are enzymes with different properties regarding kinetics and, most probably, specificity of interactions with endogenous cellular components. We have measured the diaphorase activity of Cos-7/pFNR and found that it doubles that of the control line Cos-7/pcDNA3 supporting the idea that FNR is functional and elevates basal levels of this enzymatic activity. For Fld we do not have an estimation of the expression of this protein.

MTT reduction and LPO (FOX assay) were analyzed under MV induced injury. Cells demonstrated a major reduction in viability/growth (20% of controls after 48 h incubation with 300 µM MV) and almost duplication of LPO content (Figure 3) but we could not see any protection in cells expressing FNR and Fld. These findings were surprising since both FNR and Fld have shown to protect a number of eukaryote and prokaryote organisms from MV exposure [2], [4], [5], [45]–[52]. Shimizu et al. [53] showed protection from MV damage (50–300 µM for 48 h) in Cos-7 cells overexpressing oxidized protein hydrolase. This finding does not exclude, however, that changes associated with the overexpression of FNR and/or Fld may result in the lack of protection we observed.

Other authors using MV to induce oxidative stress have observed that protective enzymes work better if their expression is directed to mitochondria when compared with cytosolic localization [54], [55]. MLS in Cos-7/pFld has not been efficiently excised but this fact did not avoid protection from H_2_O_2_. Anyway, if non-processed MLS-Fld displays lower or no function, it is possible that the achieved levels of Fld activity are insufficient to cope with MV challenge. Regarding this point van Leeuwen et al. [56] have reported that the reaction of MV radicals with Fld increases with Fld concentration and decreases when the concentration of oxidized viologen raises. In our studies we could be in the presence of low Fld (unprocessed protein) and high oxidized MV levels (high MV concentration imposed) explaining our observations.

Moreover, Figure 3A shows reduced viabilities for Cos-7/pFNR compared to the other cell lines for all the MV concentrations tested. In this sense, it has been reported that NADPH-MV diaphorase reaction of overexpressed FNR towards MV can enhance redox cycling of this compound with the consequent depletion of NADPH and augmentation of oxidative damage [4], [52]. Elroy-Stein et al. [57] reported an increased MV-mediated cytotoxity and enhancement of LPO in cells overexpressing Cu/Zn-SOD. They showed that clones possessing the highest transgene activities were less resistant to MV and that even the most resistant clones (expressing lower levels of SOD) were gradually deteriorating in the presence of low concentrations of MV for extended periods (50 µM for 48 h) [57].

In this work we have demonstrated that *P. sativum* FNR and *Anabaena* PCC 7119 Fld are able to reduce the damages caused by H_2_O_2_ but not MV in Cos-7 cells *in vitro*. This is a first study performed to evaluate these two genes as candidates for future applications in gene therapy such as amelioration of diseases and transplantation conditions for which ROS participation have been established.

## Supporting Information

Figure S1 A) Circular map of pFNR showing the principal attributes of the plasmid B) Schematic representation of hybrid FNR gene between restriction sites Hind III (red) and EcoR I (yellow) of pFNR. In green: translation initiation (ATG) and termination (TAA) codons; in blue: limit between mouse Fdrx mitochondrial localization signal (MLS) and mature portion of *Pisum sativum* FNR (FNR). C) Fusion site sequence. In red: part of Fdxr MLS (last 2 codons); in green: residual mouse Fdrx mature portion (4 codons); in black: an additional codon created by the fusion; in grey: residual *P. sativum* FNR chloroplastic localization signal (1 codon) and in blue: part of *P. sativum* FNR mature portion (first 2 codons). Underlined in black: Sac I restriction site.
(0.03 MB PDF)

Figure S2 A) Circular map of pFld showing the principal attributes of the plasmid. B) Schematic representation of hybrid Fld gene between restriction sites Hind III (red) and EcoR I (yellow) of pFld. In green: translation initiation (ATG) and termination (TAA) codons; in blue: limit between mouse Fdrx mitochondrial localization signal (MLS) and *Anabaena* Fld (Fld). C) Fusion site sequence. In red: part of Fdxr MLS (last two (2) codons); in green: residual mouse Fdrx mature portion (4 codons); in black: additional codons created by the fusion (4 codons) and in blue: part of *Anabaena* Fld (ATG, first codon). Underlined in black: Sac I restriction site.
(0.02 MB PDF)

Figure S3 A) Sequence of hybrid FNR gene between restriction sites Hind III (red) and EcoR I (yellow) of pFNR. In green: translation initiation (ATG) and stop (TAA) codons. In blue: Sac I restriction site. B) Sequence of hybrid Fld gene between restriction sites Hind III (red) and EcoR I (yellow) of pFld. In green: translation initiation (ATG) and stop (TAA) codons. In blue: Sac I restriction site.
(0.01 MB PDF)

Figure S4 Rate of growth of cell lines. Grown in culture medium without G418: Balck: Cos-7; dark grey: Cos-7/pcDNA3; light grey: Cos-7/pFNR; white: Cos-7/pFld. Grown in culture medium with G418: oblique dashed: Cos-7/pcDNA3; horizontal dashed: Cos-7/pFNR; oblique squared: Cos-7/pFld. ANOVA results: ^*^ different from cells cultured in the presence of G418; ^#^ different from all other cell lines. At 72 h (not marked in the figure to avoid confusion): 1) Cos-7/pFNR +G418 is also different from Cos-7/pcDNA3 -G418 and Cos-7/pFld +G418; and 2) Cos-7/pFNR -G418 is also different from Cos-7/pFld -G418.
(0.01 MB PDF)

Figure S5 A- Western blot analyses. FNR: detection using antibody developed against *P. sativum FNR* (C7: total extract from Cos-7; C7/p3: total extract from Cos-7/pcDNA3; C7/pFNR: total extract from Cos-7/pFNR; C7/pFld: total extract from Cos-7/pFld; C: cytoplasmic fraction of Cos-7/pFNR cells; M: mitochondrial franction of Cos-7/pFNR cells; N: nuclear fraction of Cos-7/pFNR cells). Fld: detection using antibody developed against *Anabaena* Fld (C7: total extract from Cos-7; C7/p3: total extract from Cos-7/pcDNA3; C7/pFNR: total extract from Cos-7/pFNR; C7/pFld: total extract from Cos-7/pFld; C: cytoplasmic fraction of Cos-7/pFld cells; M: mitochondrial franction of Cos-7/pFld cells; N: nuclear fractions of Cos-7/pFld cells). Positive band positions are pointed with red arrows (43.23 and 35.76 kDa for FNR; and 33.24 and 19.87 kDa for Fld). Positions of molecular weight markers are marked with blue arrows and their weights in kDa are indicated. B- Typical merges of the photomicrographs taken for co-localization studies. Staining: nuclei in blue (Hoechst 33258); mitochondria in red (MitoTracker Red); transgenic protein in green (fluorescein conjugated specific antibody).
(0.48 MB PDF)

Figure S6 Hydrogen peroxide induced cytotoxicity after (A) 2 h exposure and (B) 4 h exposure. Black: Cos-7; dark grey: Cos-7/pcDNA3; light grey: Cos-7/pFNR and white: Cos-7/pFld cells. n = 6 experiments in triplicate.
(0.01 MB PDF)
