# Supplementary figures and images for: Heterologous Ferredoxin Reductase and Flavodoxin Protect Cos-7 Cells from Oxidative Stress
Source: PLoS One. 2010 Oct 19;5(10):e13501. doi: 10.1371/journal.pone.0013501 (PMC2957446; doi:10.1371/journal.pone.0013501)

Figure S1

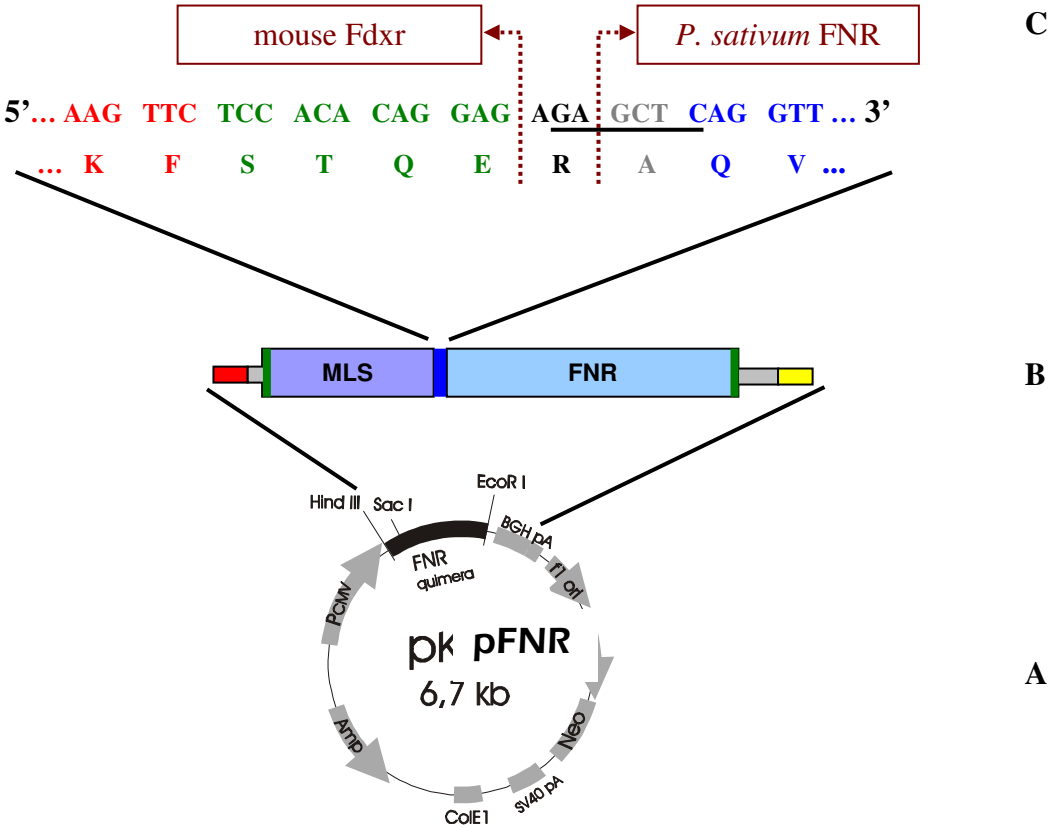

Supplement: Figure S1 — A) Circular map of pFNR showing the principal attributes of the plasmid B) Schematic representation of hybrid FNR gene between restriction sites Hind III (red) and EcoR I (yellow) of pFNR. In green: translation initiation (ATG) and termination (TAA) codons; in blue: limit between mouse Fdrx mitochondrial localization signal (MLS) and mature portion of Pisum sativum FNR (FNR). C) Fusion site sequence. In red: part of Fdxr MLS (last 2 codons); in green: residual mouse Fdrx mature portion (4 codons); in black: an additional codon created by the fusion; in grey: residual P. sativum FNR chloroplastic localization signal (1 codon) and in blue: part of P. sativum FNR mature portion (first 2 codons). Underlined in black: Sac I restriction site. (0.03 MB PDF) [file pone.0013501.s001.pdf]

Figure S2

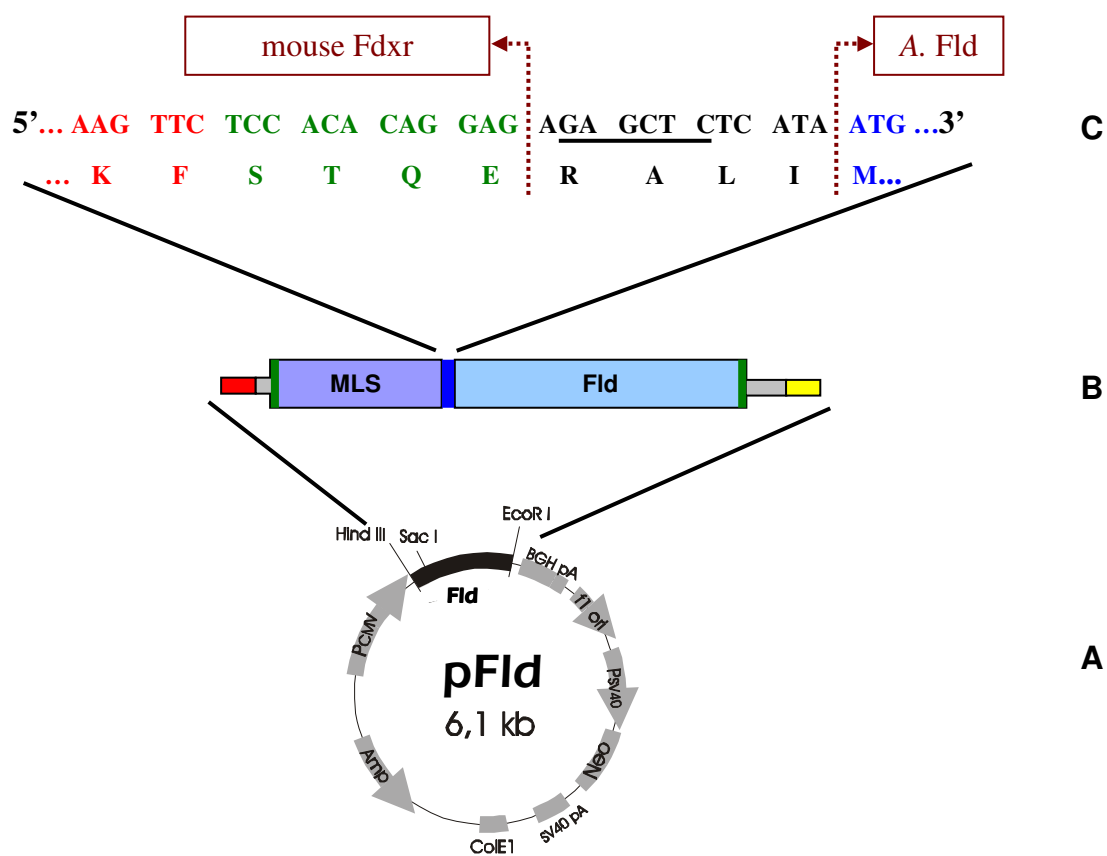

Supplement: Figure S2 — A) Circular map of pFld showing the principal attributes of the plasmid. B) Schematic representation of hybrid Fld gene between restriction sites Hind III (red) and EcoR I (yellow) of pFld. In green: translation initiation (ATG) and termination (TAA) codons; in blue: limit between mouse Fdrx mitochondrial localization signal (MLS) and Anabaena Fld (Fld). C) Fusion site sequence. In red: part of Fdxr MLS (last two (2) codons); in green: residual mouse Fdrx mature portion (4 codons); in black: additional codons created by the fusion (4 codons) and in blue: part of Anabaena Fld (ATG, first codon). Underlined in black: Sac I restriction site. (0.02 MB PDF) [file pone.0013501.s002.pdf]

Figure S4

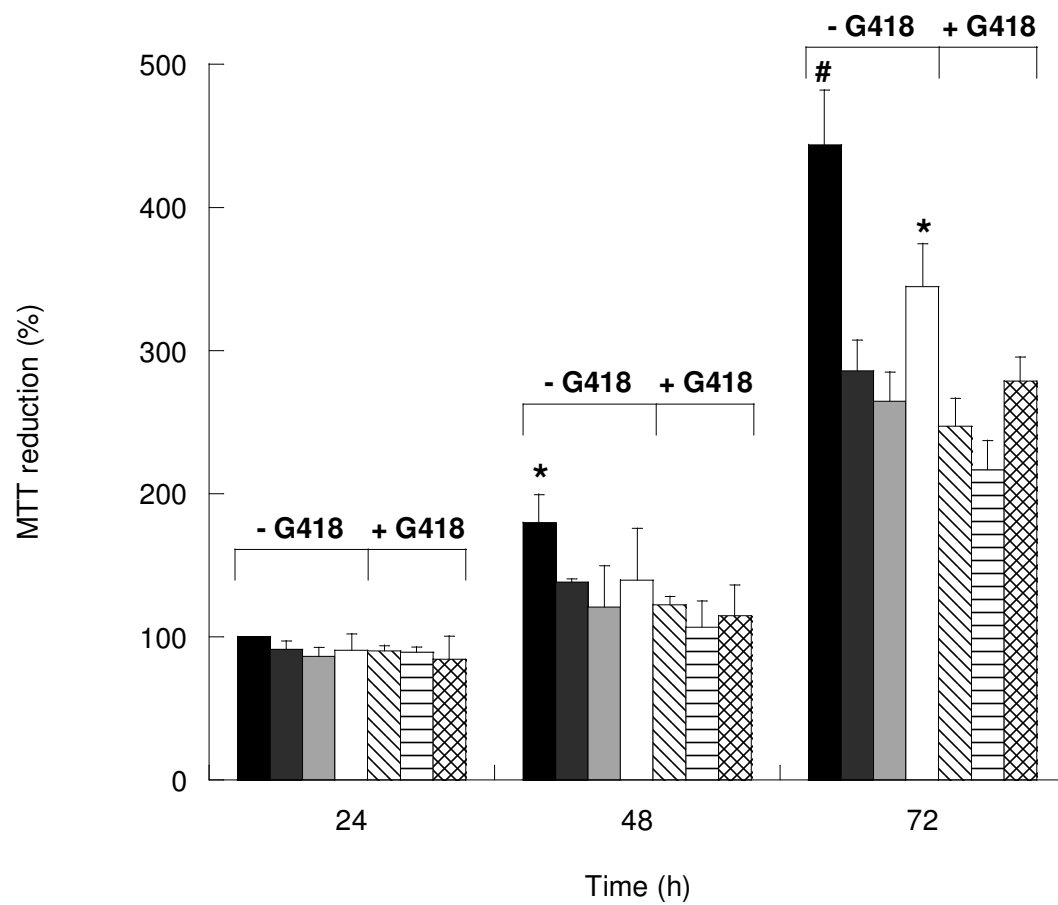

Supplement: Figure S4 — Rate of growth of cell lines. Grown in culture medium without G418: Balck: Cos-7; dark grey: Cos-7/pcDNA3; light grey: Cos-7/pFNR; white: Cos-7/pFld. Grown in culture medium with G418: oblique dashed: Cos-7/pcDNA3; horizontal dashed: Cos-7/pFNR; oblique squared: Cos-7/pFld. ANOVA results: * different from cells cultured in the presence of G418; # different from all other cell lines. At 72 h (not marked in the figure to avoid confusion): 1) Cos-7/pFNR +G418 is also different from Cos-7/pcDNA3 -G418 and Cos-7/pFld +G418; and 2) Cos-7/pFNR -G418 is also different from Cos-7/pFld -G418. (0.01 MB PDF) [file pone.0013501.s004.pdf]

Figure S5

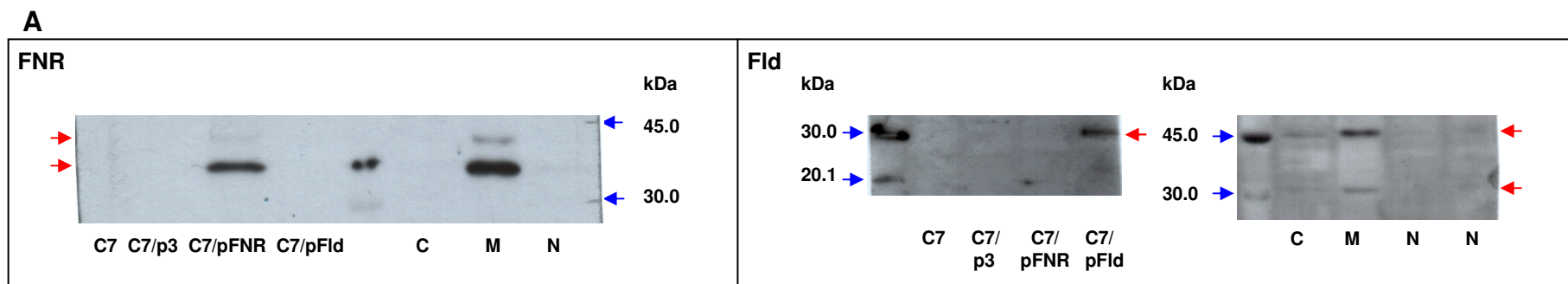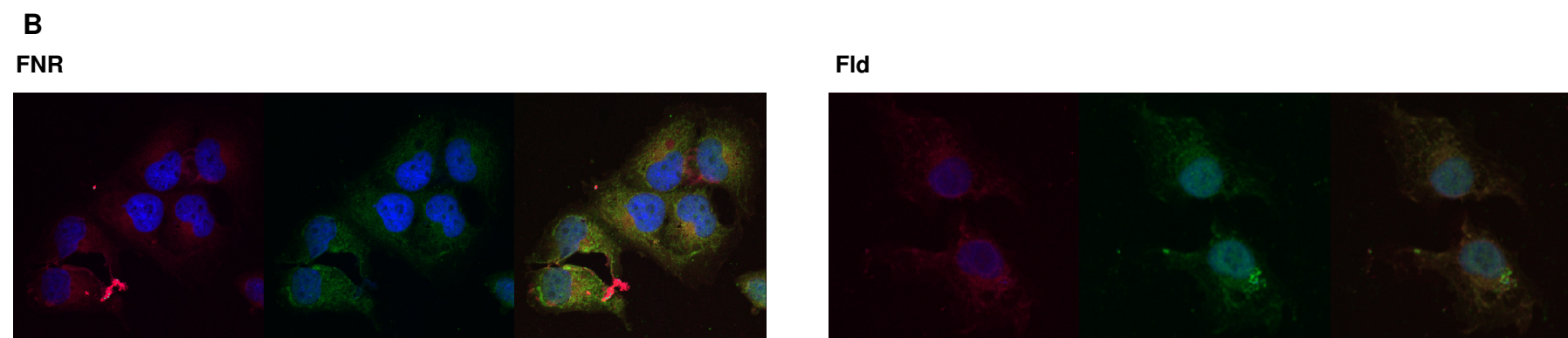

Supplement: Figure S5 — A- Western blot analyses. FNR: detection using antibody developed against P. sativum FNR (C7: total extract from Cos-7; C7/p3: total extract from Cos-7/pcDNA3; C7/pFNR: total extract from Cos-7/pFNR; C7/pFld: total extract from Cos-7/pFld; C: cytoplasmic fraction of Cos-7/pFNR cells; M: mitochondrial franction of Cos-7/pFNR cells; N: nuclear fraction of Cos-7/pFNR cells). Fld: detection using antibody developed against Anabaena Fld (C7: total extract from Cos-7; C7/p3: total extract from Cos-7/pcDNA3; C7/pFNR: total extract from Cos-7/pFNR; C7/pFld: total extract from Cos-7/pFld; C: cytoplasmic fraction of Cos-7/pFld cells; M: mitochondrial franction of Cos-7/pFld cells; N: nuclear fractions of Cos-7/pFld cells). Positive band positions are pointed with red arrows (43.23 and 35.76 kDa for FNR; and 33.24 and 19.87 kDa for Fld). Positions of molecular weight markers are marked with blue arrows and their weights in kDa are indicated. B- Typical merges of the photomicrographs taken for co-localization studies. Staining: nuclei in blue (Hoechst 33258); mitochondria in red (MitoTracker Red); transgenic protein in green (fluorescein conjugated specific antibody). (0.48 MB PDF) [file pone.0013501.s005.pdf]

Figure S6

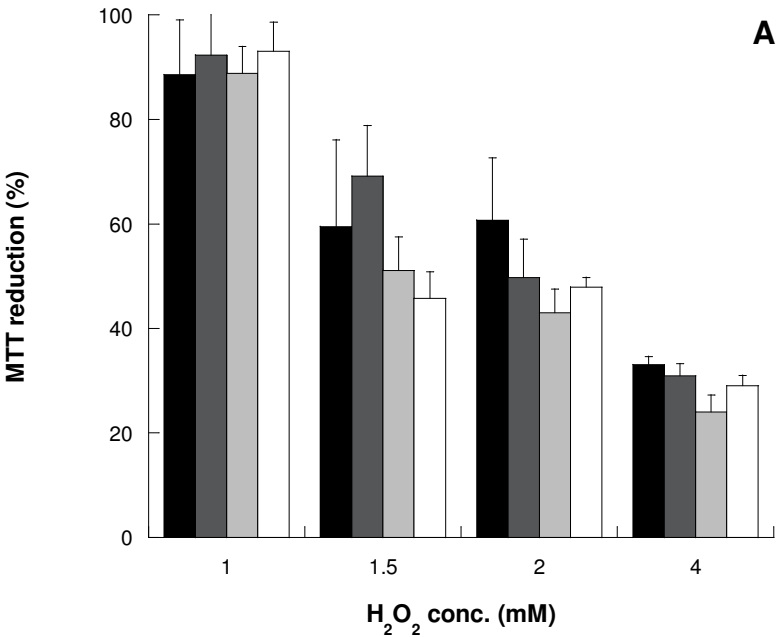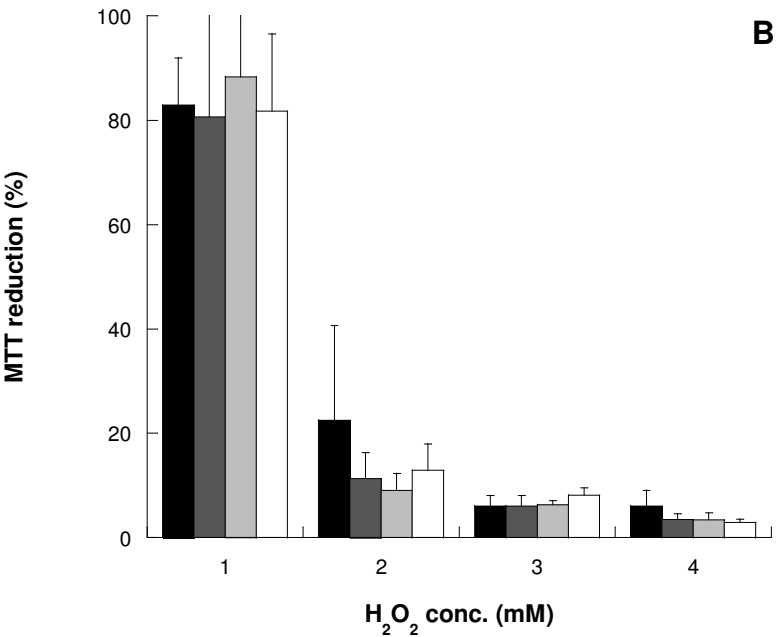

Supplement: Figure S6 — Hydrogen peroxide induced cytotoxicity after (A) 2 h exposure and (B) 4 h exposure. Black: Cos-7; dark grey: Cos-7/pcDNA3; light grey: Cos-7/pFNR and white: Cos-7/pFld cells. n = 6 experiments in triplicate. (0.01 MB PDF) [file pone.0013501.s006.pdf]
