# Supplementary material for: Heterologous Ferredoxin Reductase and Flavodoxin Protect Cos-7 Cells from Oxidative Stress
Source: PLoS One. 2010 Oct 19;5(10):e13501. doi: 10.1371/journal.pone.0013501 (PMC2957446; doi:10.1371/journal.pone.0013501)
Supplement: Figure S3 — A) Sequence of hybrid FNR gene between restriction sites Hind III (red) and EcoR I (yellow) of pFNR. In green: translation initiation (ATG) and stop (TAA) codons. In blue: Sac I restriction site. B) Sequence of hybrid Fld gene between restriction sites Hind III (red) and EcoR I (yellow) of pFld. In green: translation initiation (ATG) and stop (TAA) codons. In blue: Sac I restriction site. (0.01 MB PDF) [file pone.0013501.s003.pdf]

Figure S3

A

pFNR

AAGCTTGCTGGTTGCACTTCTCAGCC **ATG** GCT CCT CGC TGC TGG CAC TGG TGG CGC TGG TCC
GCG TGG TCT GGG CTT CGG CCG TCT CCC TCC AGG AGC ACT CCG ACC CCA GGC TTC TGC
CAG AAG TTC TCC ACA CAG GAG A**GA GCT C**AG GTT ACT ACT GAG GCA CCT GCT AAG GTA
GTA AAG CAT TCA AAG AAA CAA GAT GAA AAT ATT GTT GTG AAC AAG TTC AAA CCT AAG
GAA CCA TAC GTT GGG AGG TGT CTT CTC AAC ACA AAG ATC ACT GGT GAT GAT GCA CCT
GGT GAA ACT TGG CAC ATG GTT TTC AGC ACT GAG GGA GAG GTT CCT TAC AGA GAA GGA
CAA TCT ATT GGG ATA GTT CCT GAC GGT ATT GAC AAG AAT GGC AAG CCT CAC AAG CTG
AGA TTG TAT TCA ATT GCT AGC AGT GCC ATT GGT GAT TTT GGA GAC TCC AAA ACT GTT
TCC CTA TGT GTG AAA CGT CTT GTT TAC ACA AAC GAT GCC GGA GAA GTT GTT AAG GGA
GTT TGC TCA AAT TTC TTG TGC GAT TTG AAG CCG GGA TCA GAA GTA AAG ATT ACT GGA
CCA GTT GGT AAA GAA ATG CTT ATG CCA AAA GAT CCT AAT GCT ACT GTC ATC ATG TTG
GGA ACT GGA ACT GGA ATC GCC CCA TTT CGC TCA TTT TTA TGG AAA ATG TTC TTT GAG
AAG CAT GAA GAT TAC CAG TTC AAT GGT TTG GCA TGG CTC TTC CTA GGT GTC CCT ACA
AGC AGC TCA CTG CTT TAT AAG GAG GAA TTT GAA AAG ATG AAG GAG AAA GCA CCC GAG
AAC TTC AGG CTT GAC TTT GCT GTA AGC AGA GAG CAA GTA AAC GAT AAA GGA GAG AAA
ATG TAC ATC CAA ACA AGA ATG GCT CAA TAC GCC GAA GAG TTA TGG GAA TTA CTG AAG
AAA GAC AAC ACT TTT GTT TAC ATG TGT GGA CTG AAA GGA ATG GAA AAG GGA ATT GAT
GAC ATA ATG GTG TCA CTC GCT GCC AAA GAC GGC ATC GAT TGG ATC GAG TAC AAG AGA
ACG TTG AAG AAG GCG GAG CAA TGG AAC GTT GAA GTC TAT **TAA** TTTCTTTTCACTTGCATTG
ATAACAAGTTCCAGTTTGTGCAGCAGCTTATCTATATCACCCTCCTGTATTCTGTAGATAGATTACATTACAACC
TTGTAATTTTGGTATGACAAAAAATTATACCTTGCTTGTGAACAATATTGTTTACGGAATCGTTGCAAGAAATAA
TTTTGAGGATCATGCAATATAGTTTATCAATTTCCTGAAATTTAATTGTGAATCC**GAATTC**

B

pFld

AAGCTTGCTGGTTGCACTTCTCAGCC **ATG** GCT CCT CGC TGC TGG CAC TGG TGG CGC TGG TCC
GCG TGG TCT GGG CTT CGG CCG TCT CCC TCC AGG AGC ACT CCG ACC CCA GGC TTC TGC
CAG AAG TTC TCC ACA CAG GAG A**GA GCT C**TC ATA ATG TCA AAG AAA ATT GGT TTA TTC
TAC GGT ACT CAA ACT GGT AAA ACT GAA TCA GTA GCA GAA ATC ATT CGA GAC GAG TTT
GGT AAT GAT GTG GTG ACA TTA CAC GAT GTT TCC CAG GCA GAA GTA ACT GAC TTG AAT
GAT TAT CAA TAT TTG ATT ATT GGC TGT CCT ACT TGG AAT ATT GGC GAA CTG CAA AGC
GAT TGG GAA GGA CTC TAT TCA GAA CTG GAT GAT GTA GAT TTT AAT GGT AAA TTG GTT
GCC TAC TTT GGG ACT GGT GAC CAA ATA GGT TAC GCA GAT AAT TTT CAG GAT GCG ATC
GGT ATT TTG GAA GAA AAA ATT TCT CAA CGT GGT GGT AAA ACT GTC GGC TAT TGG TCA
ACT GAT GGA TAT GAT TTT AAT GAT TCC AAG GCA CTA AGA AAT GGC AAG TTT GTA GGA
CTA GCT CTT GAT GAA GAT AAT CAA TCT GAC TTA ACA GAC GAT CGC ATC AAA AGT TGG
GTT GCT CAA TTA AAG TCT GAA TTT GGT TTG **TAA** AAA**GAATTC**
